# *Plasmodium chabaudi* infection induces AID expression in transitional and marginal zone B cells

**DOI:** 10.1002/iid3.134

**Published:** 2016-11-28

**Authors:** Joel R. Wilmore, Alexander C. Maue, Rosemary Rochford

**Affiliations:** ^1^ Department of Microbiology and ImmunologySUNY Upstate Medical UniversitySyracuseNew YorkUSA; ^2^ Department of Immunology and MicrobiologyUniversity of ColoradoAuroraColoradoUSA

**Keywords:** activation‐induced deaminase (AID), B cells, *Plasmodium*

## Abstract

**Introduction:**

Endemic Burkitt's lymphoma (eBL) is associated with Epstein–Barr virus and repeated malaria infections. A defining feature of eBL is the translocation of the c‐myc oncogene to the control of the immunoglobulin promoter. Activation‐induced cytidine deaminase (AID) has been shown to be critical for this translocation. Malaria infection induces AID in germinal center B cells, but whether malaria infection more broadly affects AID activation in extrafollicular B cells is unknown.

**Methods:**

We either stimulated purified B cells from AID‐green fluorescence protein (GFP) reporter mice or infected AID‐GFP mice with *Plasmodium chabaudi*, AID fluorescence was monitored in B cell subsets by flow cytometry.

**Results:**

In vitro analysis of B cells from these mice revealed that CpG (a Toll‐like receptor 9 ligand) was a potent inducer of AID in both mature and immature B cell subsets. Infection of AID‐GFP mice with *Plasmodium chabaudi* demonstrated that AID expression occurs in transitional and marginal zone B cells during acute malaria infection. Transitional B cells were also capable of differentiating into antibody secreting cells when stimulated in vitro with CpG when isolated from a *P. chabaudi*‐infected mouse.

**Conclusions:**

These data suggest that *P. chabaudi* is capable of inducing AID expression in B cell subsets that do not participate in the germinal center reaction, suggesting an alternative role for malaria in the etiology of eBL.

## Introduction

Antigen receptor editing and isotype switching are found uniquely in B cells and require the activity of activation‐induced cytidine deaminase (AID). The ability of B cells to fine tune their antigen receptors through somatic hypermutation (SHM) and class‐switch recombination (CSR) makes antibody‐mediated immunity broadly reactive and highly effective 1. The downsides of manipulating genomic DNA are the increased risk of off‐target mutations and lymphomagenesis. The activity of AID has been implicated in several B cell lymphomas including endemic Burkitt's lymphoma (eBL). The activity of AID is necessary to induce c‐myc translocations commonly seen in certain B cell lymphomas and the over‐activity of AID is sufficient to lead to malignancy in mice 2, 3. Endemic BL is tightly linked to malaria and EBV infection; however, only recently has there been a direct link between *Plasmodium* infection and AID‐induced translocations 4. Robbiani et al. 4 implicate the ability of *Plasmodium* to induce prolonged germinal center reactions as the main culprit, but do not rule out the possibility that *Plasmodium* can induce AID expression in B cells outside of germinal center reactions. The role of *Plasmodium* as a broadly acting B cell stimulator on immature B cell populations has not been studied in depth and may represent an additional pathway to lymphomagenesis.

Germinal centers are the primary location where B cells undergo antibody editing by SHM and CSR 5. These processes require the activity of the enzyme AID. Recently, there have been reports of low levels of AID expression in cells outside the germinal centers during extrafollicular antibody responses and in immature B cells 6, 7, 8, 9. Additionally, in vitro studies using toll‐like receptor (TLR)‐9 stimulation have demonstrated that human transitional B cells can undergo AID‐dependent SHM 10. The restriction of AID to germinal centers is important because within this unique microenvironment signals that regulate proliferation and apoptosis are readily available to constrain aberrant B cell activation 11. Therefore, AID expression outside of germinal centers may potentially lead to an increased risk of mutations and the potential for developing malignancies in the absence of regulation.

Endemic BL is a poly‐microbial disease that in most cases requires the presence of both Epstein–Barr virus (EBV) and *P. falciparum* for malignant transformation of B cells 12. EBV is capable of rescuing cells with constitutive c‐myc expression from apoptosis, but the high prevalence of EBV in the human population suggests that additional factors are needed to induce lymphoma development 13. Recent evidence using mouse models has shed light on the potential of *Plasmodium* to induce AID expression in germinal centers that is capable of leading to genomic instability and c‐myc translocations 4. However, the role of *Plasmodium* in stimulating cells outside of the germinal center reaction has not been studied in detail. This distinction is important as *Plasmodium* can lead to polyclonal activation of B cells by multiple direct and indirect mechanisms. For example, *P. falciparum* erythrocyte membrane protein (PfEMP)‐1 is capable of directly activating B cells by binding to CD36 and/or IgM 14, 15. The *Plasmodium* metabolic breakdown product, hemozoin, bound to DNA can lead to polyclonal activation of B cells through TLR9 16. Stimulation by these *Plasmodium* B cell activators is not restricted to a certain cell niche and all B cells have the potential to be exposed during infection.

In this study, we tested the hypothesis that malaria is capable inducing aberrant AID activity in extrafollicular B cells by using the *P. chabaudi* mouse model of blood stage malaria. *P. chabaudi* is a natural rodent pathogen that is similar to *P. falciparum* in its cytoadherence properties and CD36 binding 17. We utilized transgenic AID‐GFP mice on a C57BL/6 background to determine which B cell subsets expressed AID in response to infection with *P. chabaudi* 18.

## Results

### In vitro CpG stimulation leads to AID expression in both mature and immature B cell subsets

**Figure 1:**
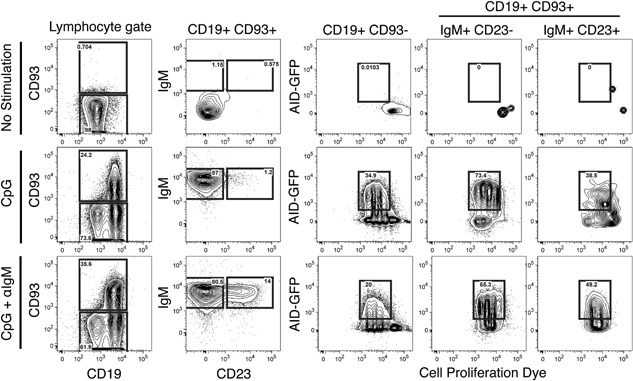
CpG stimulation leads to AID expression in mature, and T1 B cells and CpG+anti‐IgM resulted in the expression of AID in T2 B cells. B cells enriched by negative selection from AID‐GFP mice were cultured in the presence of media alone, CpG, or CpG+anti‐IgM for 3 days. Populations of mature, T1 and T2 B cell subsets were analyzed for their expression of GFP. Representative flow cytometry showing the mature (CD19+CD93−) and immature (CD19+CD93+) B cells on the left panel. Transitional types 1 and 2 B cells were differentiated in the second column by IgM and CD23 expression (T1 = IgM+, CD23− and T2 = IgM+, CD23 +). The right three columns show AID‐GFP expression for the mature B cells (CD19+ CD93−), T1 cells, and T2 cells compared to a cell proliferation dye (*n* = 3 in two independent experiments).

In vitro studies of human immature B cells have shown that CpG stimulation through TLR9 signaling is capable of inducing AID activity 10. We wanted to determine whether TLR9 signaling in B cells induces AID in a mouse model system using an AID‐GFP transgenic reporter mouse 18. To do this, we enriched splenic B cells by negative selection. Following B cell enrichment, we obtained a heterogeneous population of B cell subsets that included both immature and mature B cells (CD19 + CD93+ and CD19 + CD93−, respectively). We then stained the cells with a proliferation dye stimulated these B cells with CpG and anti‐IgM F(ab′)2 fragments to determine whether CpG alone, or in conjunction with BCR crosslinking, activated AID expression. AID expression was then measured in immature and mature B cell subsets after 3 days stimulation by flow cytometry. When cultured in the absence of stimulation the immature B cells (CD19+, CD93+) made up <1% of total cells in as little as 1 day in culture (data not shown) and remained absent after 3 days (Fig. 1). Additionally, unstimulated B cells expressed low levels of CD19 and were almost completely AID negative (Fig. 1). Stimulation with anti‐IgM alone resulted in the same phenotype as control cells for all parameters analyzed (data not shown). After 3 days in culture, CpG‐stimulated B cells comprised two populations of CD19^high^ and CD19^low^ cells. The CD19^high^ population consisted of the blasting cells that had undergone division and included both immature and mature B cells (Fig. 1). The majority of the CpG‐stimulated immature B cells were AID+ and had an IgM+CD23− or transitional type 1 (T1) phenotype (Fig. 1). When CpG stimulation was combined with BCR cross‐linking with anti‐IgM, there were similar levels of AID expression as CpG alone. Interestingly, the phenotype of the immature B cells following treatment with both anti‐IgM and CpG included both T1 and IgM+ CD23+ transitional type 2 (T2) B cells (Fig. 1). These data demonstrate that CpG has the ability to induce AID expression in vitro in both mature and T1 B cells, and that anti‐IgM treatment in conjunction with CpG is capable of leading to persistence of AID expressing T2 B cells.

### *P. chabaudi* infection is capable of inducing AID expression in transitional B cell subsets

**Figure 2:**
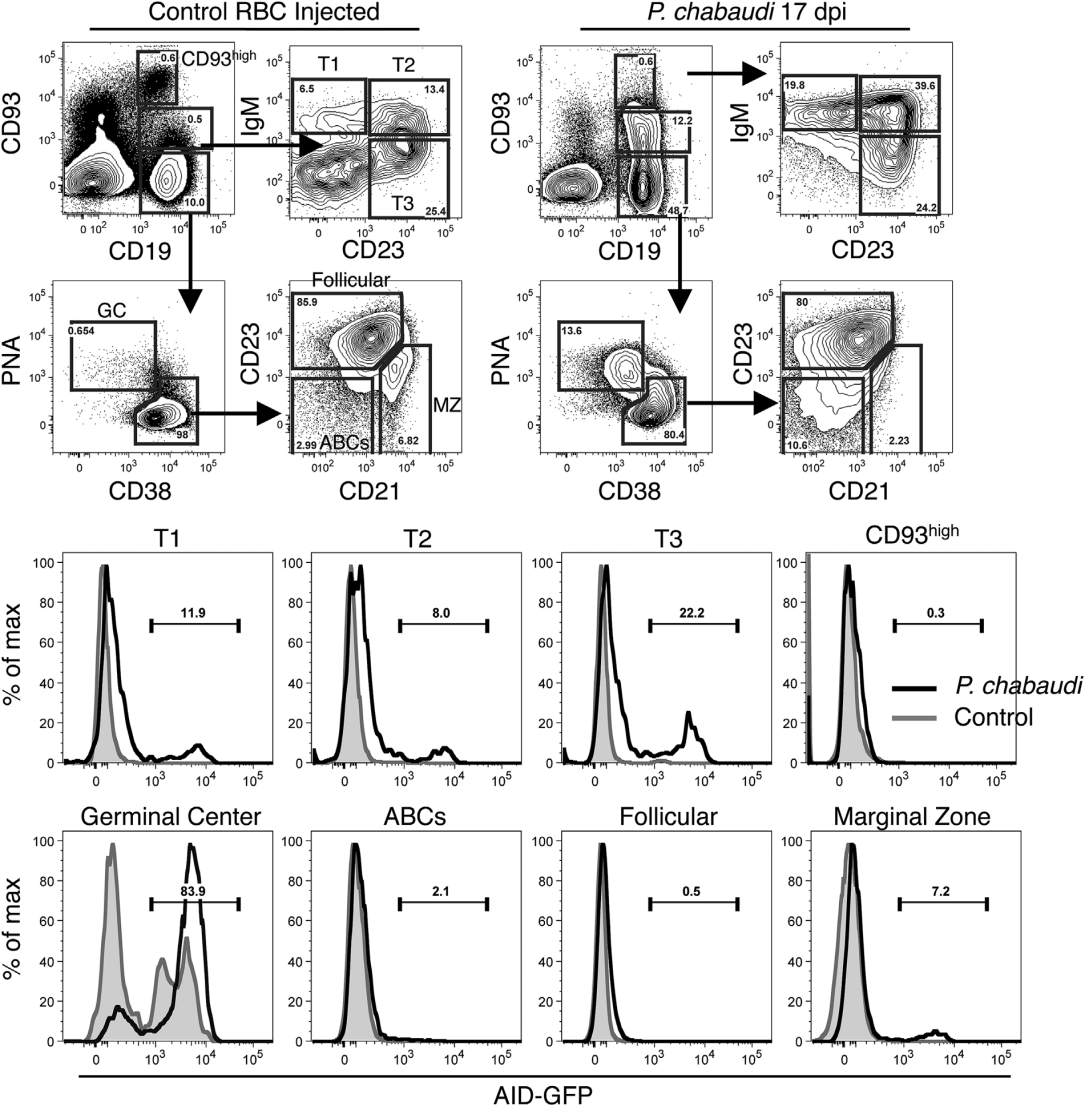
The levels of immature B cells decrease dramatically at 17 days post *Plasmodium chabaudi* infection express AID. AID‐GFP mice were infected with *P. chabaudi* or given a control injection of uninfected RBC and sacrificed at 17 dpi. Spleens were removed and analyzed by flow cytometry. Representative flow cytometry plots demonstrating the gating strategy for each of the B cell subsets are shown. The left panel is differentiating the mature (CD19+, CD93−) and immature (CD19+, CD93+), and CD93^high^ B cells based on expression of CD19 and CD93. Transitional B cells subsets are gated into T1 (IgM+, CD23−), T2 (IgM+, CD23+), and T3 (IgM−, CD23+) from the immature B cell subset. Germinal center B cells (PNA^high^, CD38−) and the rest of the mature B cells are further analyzed to determine the follicular (PNA−, CD38+, CD23+, CD21+) and marginal zone (PNA−, CD38+, CD23^int^, CD21^high^) B cells. The bottom panel contains representative flow cytometry histograms demonstrating the expression of AID in each B cell subset (*P. chabaudi* infected in black, RBC control in gray histograms) (*n* = 3 in two independent experiments).

Previous studies performed with AID‐GFP mice demonstrated that AID expression was restricted to germinal center B cells following injection with NP‐CGG and alum, a model antigen and adjuvant commonly used to elicit a humoral immune response 19. *P. chabaudi* is a productive infection with a higher antigen load and inflammatory response than a model antigen injection such as NP‐CGG. Therefore, we used AID‐GFP mice in the *P. chabaudi* model to determine whether AID can be expressed in B cells outside of the germinal center. We performed RT‐qPCR on sections of whole spleen at 6, 12, 17, 24, and 30 days post‐infection (dpi) with *P. chabaudi* and found that the peak of AID expression occurred at 17 dpi (data not shown). The 17‐day time point was then used to determine the percentage of AID positive cells within the T1, T2, T3, marginal zone, follicular, and germinal center B cell subsets by multivariate flow cytometry. At 17 dpi, we found dramatic differences in splenic B cell subset distribution, including a significant decrease in immature and marginal zone B cells, and a large expansion of the germinal center B cell population (Fig. 2), which were consistent with previously reported studies 20. We determined the percentage of AID expressing cells from the immature T1 (IgM+CD23−), T2 (IgM+CD23+), and T3 (IgM‐CD23+) B cell subsets in mice 17 dpi or control mice injected with uninfected RBC. All three immature B cell populations had detectable AID expression 17 days after *P. chabaudi* infection, and the control mice had little or no AID expressed (Fig. 2). In addition to the CD93+ population of immature B cells, the *P. chabaudi*‐infected mice had an additional CD93^high^ population of cells that were AID− (Fig. 2). Germinal center B cells (CD38‐PNA^high^) were greatly expanded during *P. chabaudi* infection and >80% expressed AID (Fig. 2). The control germinal center B cells also expressed AID, but at a lower frequency (Fig. 2). During acute *P. chabaudi*, the percentage of marginal zone B cells decreased dramatically 19; our study confirmed this finding and demonstrated the marginal zone B cell population that remained included cells that expressed AID (Fig. 2). Follicular B cells in both the *P. chabaudi*‐infected and control mice did not express AID (Fig. 2). The expansion of CD23− CD21− B cells is consistent with reports of age associated B cells (ABCs) that expand during infection 21, 22. Interestingly, the ABCs had low expression of AID despite their induction during infection. These data demonstrate that during *P. chabaudi* infection, the expression of AID is not restricted to the germinal center B cells, but is also expressed in transitional and marginal zone B cells.

### Transitional B cells isolated from *P. chabaudi‐*infected mice have an increased production of plasma cells

**Figure 3:**
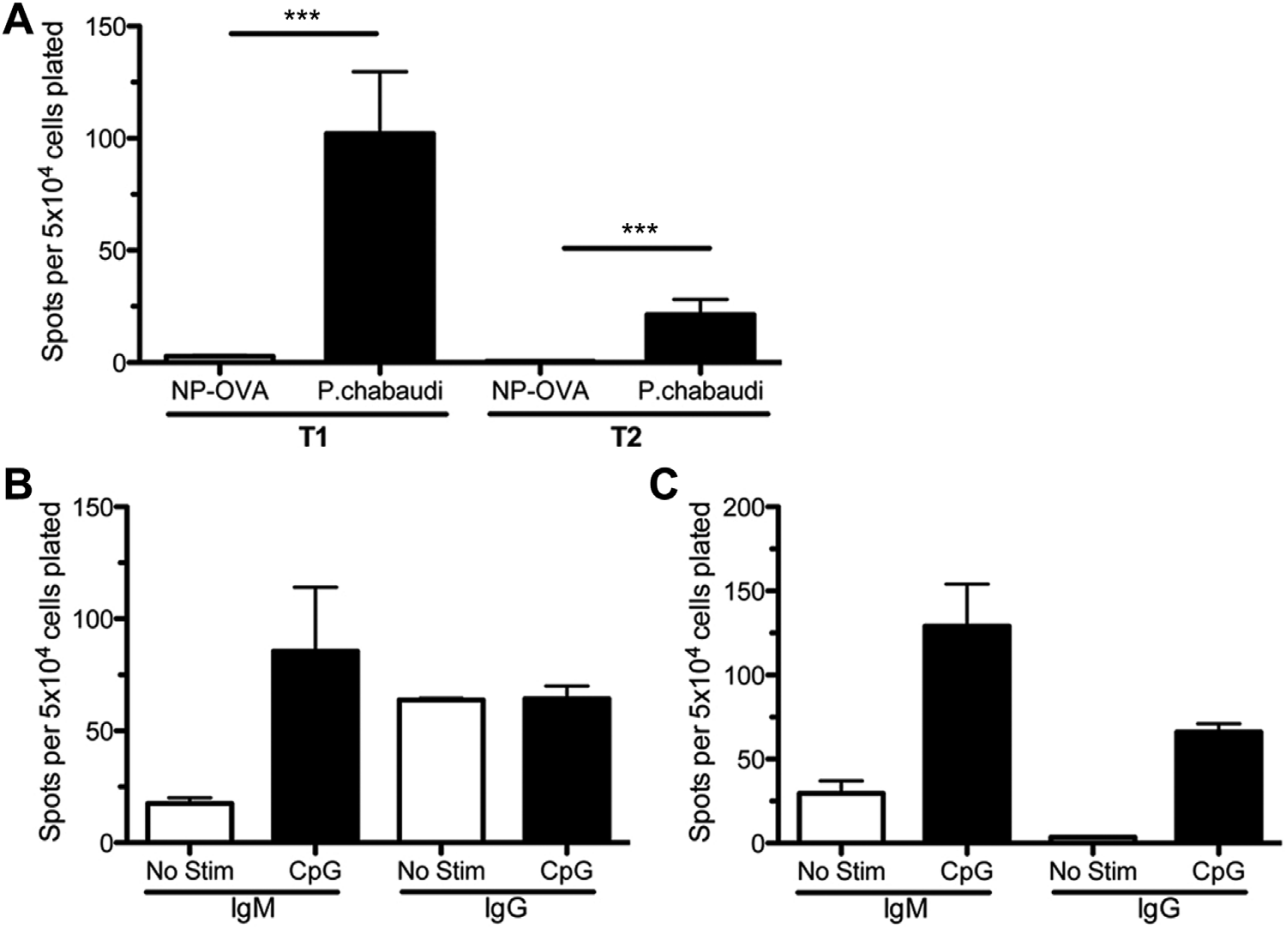
Transitional B cells secrete antibody when isolated from *Plasmodium chabaudi‐*infected mice, but not control mice. (A) ELISpot plates coated with anti‐mouse IgM and IgG were plated with sorted T1 (CD19+, CD93+, IgM+, CD23−, CD138−) and T2 (CD19+, CD93+, IgM+, CD23+, CD138−) B cells from *P. chabaudi‐*infected mice or control NP‐OVA‐injected mice and spots were detected with a combination of anti‐IgM and −IgG antibodies. T1 and T2 B cells from *P. chabaudi‐*infected mice produced significantly more spots than cells from control mice (*n* = 3–5). (B) T1 B cells from uninfected mice were cultured with or without CpG on ELISpot plates coated with either anti‐IgM or −IgG antibodies and there was no significant difference between the groups for IgM or IgG secreting cells (*n* = 2). (C) T2 B cells treated with CpG had higher numbers of IgG secreting cells and IgM secreting cells in response to CpG. *P < 0.05, **P < 0.01.

To determine whether the polyclonal activation of transitional B cells by *P. chabaudi* is capable of leading to differentiation into plasma cells, we performed ELISpot assays on sorted splenocytes. Mice were either infected with *P. chabaudi* or given an injection of NP‐OVA and alum. T1 and T2 B cells were isolated from both sets of mice at 17 dpi by flow cytometry based cell sorting. Immature B cells were defined as CD138−, CD19+, CD93+, and IgM+ while T1 and T2 B cells were differentiated by CD23 −/+ expression respectively. Sorted T1 and T2 B cells (purity T1>98% and T2 ≈ 75%) were plated on ELISpot plates coated with either polyclonal anti‐IgM or anti‐IgG and spots were developed with the corresponding alkaline phosphatase‐conjugated polyclonal antibodies. There were significantly more antibody secreting cells in the wells with T1 and T2 B cells isolated from *P. chabaudi*‐infected mice compared to mice injected with NP‐OVA and adjuvant (Fig. 3A). As the cells were cultured in media with no other stimulation, T1 and T2 B cells were responding to stimuli received in vivo, thus leading to antibody secretion in the ELISpot plates. To determine whether CpG was capable of leading to plasma cell differentiation of T1 and T2 B cells from naïve mice, we stimulated sorted transitional B cell populations in vitro for 3 days in culture in ELISpot plates. The plates were coated with anti‐IgM or anti‐IgG and cells were plated in the presence or absence of CpG. T1 B cells differentiated into IgM plasma cells at a higher frequency when stimulated with CpG, but differentiation to IgG secreting plasma cells was not changed between CpG and untreated wells (Fig. 3B). T2 B cells had an increased frequency of both IgM and IgG secreting plasma cells (Fig. 3C). The presence of spots in the wells that did not receive stimulation suggests that the flow staining and sorting could have stimulated the cells to some degree making it important to compare directly between the CpG and no stimulation for each group. These data demonstrate that transitional B cells are capable of differentiating to plasma cells in response to malaria infection in vivo and CpG stimulation in vitro.

## Discussion

The present study set out to determine whether *Plasmodium* infection induces AID activity in B cell subsets that reside outside of the germinal center. In vitro treatment of B cells with CpG revealed that AID was expressed in both mature and immature B cell subsets following TLR9 stimulation. In vivo experiments revealed that infection of mice with *P. chabaudi* leads to dramatic effects on B cell subset distribution as well as the expression of AID in transitional and marginal zone B cells. These data show AID expression in immature B cells can be activated by TLR9 stimulation or by *P. chabaudi* infection, suggesting a potential source of B cells that can become malignant through aberrant AID activation.

Transitional B cells typically do not maintain their immature phenotype or survive in culture without survival signals or growth factors. A previous study has shown that immature B cells express TLRs and can respond to various pathogen‐associated molecular patterns (PAMPs) by differentiating into marginal zone B cells 23. In our study, we stimulated a mixed population of mature and immature B cells with CpG alone or in conjunction with anti‐IgM. Immature B cells in culture with media alone either matured/differentiated or underwent apoptosis, but in the presence of CpG, the T1 B cells expanded. Stimulation with CpG alone led to a lack of T2 immature B cells without the presence of BCR cross‐linking by IgM F(ab′)2 fragments. In human studies, AID expression induced by CpG in immature B cells coincided with SHM of variable genes 10. This demonstrates that immature B cells can respond to innate stimuli‐like CpG with robust activation and the potential to affect the immune response. *Plasmodium‐*associated proteins such as PfEMP‐1 have been shown to bind to the BCR and therefore *P. chabaudi* could have a dual effect on immature B cells by stimulating with via CpG binding TLR9 and BCR cross‐linking. This represents a mechanism by which *P. chabaudi* interacts directly with B cells leading to AID expression in immature B cells subsets.

B cells that give rise to Burkitt's lymphoma are thought to be post‐germinal center B cells because of the presence of SHMs and activation markers 24. However, the recent evidence demonstrating SHM in immature B cells activated in vitro retracts from this hypothesis because the conclusion that SHM can only be found in post‐germinal center B cells is not necessarily the case 15. Previously, we have linked AID expression in PBMC to infection with *Plasmodium* and EBV in children at risk for eBL 25. There is no mouse model for eBL as a poly‐microbial disease and therefore it is difficult to recapitulate the disease as it occurs in humans. Therefore, we focused on the association of eBL with malaria to determine whether *P. chabaudi* is capable of inducing AID expression in B cell subsets outside the germinal center. Infection with *P. chabaudi* resulted in AID expression at 17 dpi in all three transitional B cell subsets and marginal zone B cells, but not follicular B cells. Interestingly, AID expression did not occur at appreciable levels early in infection when there was high parasitemia (peak parasitemia ∼10 dpi) and presumably a higher concentration of antigen and PAMPs that could activate B cells.

Transitional B cell levels were significantly reduced during *P. chabaudi* infection, which contradicts our in vitro data showing an expansion of the T1 B cells following stimulation with CpG. There are several factors that could contribute to this discrepancy between in vitro and in vivo experiments. During the in vivo *P. chabaudi* experiment, the splenocytes were not harvested until 17 dpi, which could lead to an immature B cell pool that has already differentiated or undergone apoptosis following extended activation. The immature B cells in the CpG culture experiment were harvested at 3 days post‐treatment and only have an effect on the less mature T1 population. Another factor that could lead to the decrease in transitional B cells is the shift in bone marrow lymphopoiesis to myelopoiesis, leading to fewer B cells that egress from the bone marrow 26. The appearance of the CD93^high^ cells in the spleen is further evidence of altered lymphopiesis and is likely the result of B cell precursors developing in the spleen in response to infection. Additionally, the mechanism of the interaction between *P. chabaudi* and an immature B cells is not necessarily through TLR9 signaling, and could be the result of an indirect mechanism. Further studies are required to determine the mechanism responsible for activating AID expression in immature B cells during *P. chabaudi* infection.

Previous studies of human immature B cells stimulated in vitro have shown that transitional B cells can terminally differentiate to plasma cells in response to CpG stimulation 27. Transitional B cells directly becoming plasma cells has been proposed as a potential mechanism by which the host rapidly boosts the natural antibody levels in response to a pathogen 27. Therefore, a precedent exists for the differentiation of plasma cells directly from transitional B cells seen in cells sorted from *P. chabaudi* infected mice. Also, in vitro stimulation of purified T1 B cells with CpG demonstrated that TLR9 signaling induced plasma cell differentiation in our mouse model. These data support the previous findings of immature B cell TLR9 stimulation leading to plasma cell production that was observed in human cells 27. The percentage of transitional B cells in the spleen decreases dramatically during *P. chabaudi* infection. Defining subsets of cells based on phenotype alone is problematic during *P. chabaudi* infection due to the presence of immune altering stimuli and disruption of splenic architecture. However, there are several pieces of data that suggest that these transitional‐like B cells are T1 B cells that are differentiating to become plasma cells. The ability of sorted T1 B cells, defined as CD19+, CD93+, IgM+, CD23−, and CD138−, which have been isolated from *P. chabaudi* infected mice to secrete immunoglobulin after 3 days in culture, suggests that these cells are undergoing a differentiation. Further kinetics analysis of T1 to plasma cell differentiation, as determined by ELISpot assay, will provide better insight into the activation of these cells. It is also possible that *P. chabaudi* is inducing a polyreactive response similar to those seen in other infectious models 28. If *P. chabaudi* is inducing T1 B cells to express AID and differentiate to plasma cells, then that could be a mechanism of malaria‐induced lymphoma development and reduced long‐term immunity.

In summary, *P. chabaudi* infection is capable of inducing transitional B cell activation and expression of AID. The induction of AID in T1 B cells also occurs in response to CpG stimulation in vitro, suggesting a potential role of TLR signaling in the *P. chabaudi*‐induced response. The exact mechanism of *P. chabaudi* interaction with immature B cells is unknown. T1 B cells may also be differentiating directly into plasma cells in response to CpG and/or *P. chabaudi*, suggesting a potential mechanism leading to the lack of immature B cells 17 days post *P. chabaudi* infection. These findings have implications for the etiology of eBL, demonstrating that malaria can induce AID expression in cells that do not participate in the germinal center that could potentially lead to mutations and lymphomagenesis.

## Materials and Methods

### Animals/Ethics Statement

Male C57BL/6J mice (8–10‐week‐old) were obtained from Jackson Laboratories or bred at SUNY Upstate Medical University. AID‐GFP transgenic mice, a gift of Rafael Casellas, Genomics & Immunity, NIAMS‐NCI, NIH, were bred at SUNY Upstate Medical University. Animals were maintained under specific pathogen‐free conditions. All research involving animals has been conducted as required by the Animal Welfare Act, Public Health Service Guidelines and New York State regulations with respect to husbandry, experimentation, and welfare. Protocols used for animal experiments were approved by the SUNY Upstate Medical University Committee on the Humane Use of Animals.

### Experimental *P. chabaudi* infection and antigen injections

*P. chabaudi chabaudi* AS parasitized red blood cells (pRBCs) were stored at −80°C in glycerolyte solution until use. Prior to experimental use, frozen‐infected red blood cells were thawed and injected into donor mice for one passage to propagate *P. chabaudi*. Naïve C57BL/6 mice were injected IP with 5 × 10^5^ pRBCs. Parasitemia was determined at selected days post‐infection by microscopic examination of Giemsa‐stained thin blood smears. C57BL/6 were injected IP with 50 µg of 4‐hydroxy‐3‐nitrophenyl acetyl conjugated to ovalbumin (NP‐OVA) adsorbed onto alum (Biosearch Technologies, Petaluma, CA, USA) or prepared as described previously 29.

### B cell isolation and flow cytometry

Spleens were collected from infected and/or immunized mice at various time‐points post‐infection. Spleens were mechanically dissociated in phosphate buffered saline (PBS) and passed through a 70 µm nylon mesh screen, yielding a single cell suspension. RBCs were lysed by addition of ammonium chloride lysis buffer. Total and live cells were enumerated using trypan blue (Sigma, St. Louis, MO, USA) exclusion with a hemocytometer. B cells were isolated using a magnetic bead‐based B cell negative selection kit according to the manufacturers protocol (Miltenyi Biotec, San Diego, CA, USA) and stained with Cell Proliferation Dye eFluor450 (eBioscience, San Diego, CA, USA). B cells were cultured in complete media (RPMI, 10%FBS, l‐Gutamine, Pen/Strep). Stimulation was performed by adding 10 ng/ml CpG (InvivoGen, San Diego, CA, USA), 10 ng/ml anti‐IgM F(ab′)2 (Jackson ImmunoResearch, West Grove, PA, USA), or the combination for 3 days. Flow cytometry was performed as previously described 30. In brief, cells were stained in PBS containing 1% BSA and 0.1% sodium azide for flow cytometry or in PBS with 1% BSA for cell sorting on ice with monoclonal antibodies against the following cell surface molecules: AA4, B220, IgM, CD38 (eBiosciences), CD19, CD23, CD21, (Biolegend, San Diego, CA, USA), and CD138 (BD Biosciences). Fc receptors were blocked using 2.4G2 monoclonal antibody. Peanut agglutinin‐FITC (PNA‐FITC) (Vector Laboratories, Burlingame, CA, USA) was used to identify germinal center phenotype B cells. Following staining, cells were analyzed using a LSRII analyzer (Becton Dickinson, Franklin Lakes, NJ, USA) capable of 11‐ color analysis or sorted on a FACS Aria II cell sorter (Becton Dickinson). Data were analyzed using FlowJo software (Tree Star, Ashland, OR, USA).

### ELISpot/ELISA

Multiscreen‐HA plates (Millipore, Billerica, MA) were coated with anti‐mouse IgM, and IgG (H+L) (Jackson ImmunoResearch). Sorted T1 or T2 B cells were added to the Multiscreen plates starting with 10^5^ cells followed by twofold serially dilutions and incubated for 72 h at 37°C in 5% CO_2_ incubator. Cells were removed and goat anti‐mouse IgM and Ig (H+L) AP‐conjugated antibody (Southern Biotech, Birmingham, AL, USA) were added and incubated for 2 h at 37°C. Spots were developed with SigmaFast BCIP/NBT (Sigma) and scanned and counted using an ImmunoSpot Analyzer (Cellular Technology Ltd., Shaker Heights, OH, USA). Statistical analysis was done using a Student's *t*‐test.

## Author contributions

JW, AM, and RR designed the experiments. JW and AM performed the experiments and analyzed the data. JW and RR wrote the manuscript and all authors agreed with the final version of the manuscript.

## Conflict of Interest

None declared.
